# Unplanned 6-month readmissions and in-hospital outcomes for the management of acute intermediate- and high-risk pulmonary embolism: comparisons of endovascular interventions

**DOI:** 10.1186/s42155-026-00752-1

**Published:** 2026-08-12

**Authors:** Waseem Wahood, Hayden Hofmann, Omar Seyam, Deepak Iyer, Peter Monteleone, Jonathan D. Paul, Osman Ahmed

**Affiliations:** 1 Department of Interventional Radiology, Jackson Memorial Hospital, University of Miami Miller School of Medicine, Miami, FL USA; 2 Department of Interventional Radiology, David Geffen School of Medicine at UCLA, Los Angeles, CA USA; 3 Department of Medicine, Lenox Hill Hospital, New York, NY USA; 4 Department of Diagnostic, Molecular, and Interventional Radiology, Icahn School of Medicine at Mount Sinai, New York, NY USA; 5 UT Austin Dell School of Medicine, Ascension Texas Cardiovascular, Austin, TX USA; 6 Department of Interventional Cardiology, University of Chicago, Chicago, IL USA; 7 Department of Radiology, University of Utah, Salt Lake City, UT USA

**Keywords:** Pulmonary embolism, Thrombectomy, Catheter-directed thrombolysis, ICH, Unplanned readmissions, Comparison

## Abstract

**Background:**

To compare catheter directed thrombolysis (CDT) and endovascular mechanical thrombectomy (MT) for acute intermediate-risk and high-risk pulmonary embolism (PE) with respect to unplanned 6-month readmissions and in-hospital outcomes using a national database.

**Methods:**

The NRD was queried from 2016 to 2022 for adult patients with acute nonseptic PE who received either CDT or MT. Inverse probability weighted regression adjustment was used to compare CDT and MT with regard to in-hospital mortality, unplanned 6-month readmissions, discharge other than home (DOTH), total cost as well as gastrointestinal bleeding (GIB), intracranial hemorrhage (ICH), blood transfusion at index admission, and 6-month readmission. Results are depicted as average treatment effect (ATE).

**Results:**

A total of 28,376 patients were identified; 18,168 (64%) underwent CDT and 10,208 (36%) underwent MT. There was a total of 3,945 patients with high risk PE (13.9%). Compared to CDT, the ATE of MT was higher with regards to in-hospital mortality (ATE: 0.007; *p* = 0.048), DOTH (ATE: 0.031; *p* < 0.001), ICH (ATE: 0.006; *p* < 0.001), total cost at index admission (ATE: 0.118; *p* < 0.001), GIB at 6 months (ATE: 0.004; *p* = 0.023), blood transfusions at 6 months (ATE: 0.006; *p* = 0.017) as well as death at final follow-up (ATE: 0.009; *p* = 0.025). The ATE was similar between CDT and MT with regard to GIB (*p* = 0.097) at index admission as well as unplanned 6-month readmission (*p* = 0.10), ICH (*p* = 0.11), and recurrent PE (*p* = 0.39) at 6 months.

**Conclusion:**

MT was associated with higher rates of mortality, DOTH, ICH, and total cost at index admission as well as GIB, blood transfusions, and mortality at 6 months. MT was associated with similar rates of GIB at index admission, as well as unplanned readmission, ICH, and recurrent PE at 6 months.

**Supplementary Information:**

The online version contains supplementary material available at 10.1186/s42155-026-00752-1.

## Introduction

Acute pulmonary embolism (PE) is a major cause of morbidity and mortality and the third leading cause of cardiovascular death [1]. Catheter directed thrombolysis (CDT) was utilized to minimize bleeding complications through localized delivery of thrombolytics using lower dosages of thrombolytics over a prolonged duration. CDT is currently used primarily in patients with intermediate-high and high-risk pulmonary embolism [2]. These therapies were not developed to replace systemic thrombolysis but rather to provide alternative reperfusion strategies using localized thrombolytic delivery or large-bore aspiration systems adapted from technologies long used in other vascular territories.

Systemic thrombolysis and CDT have historically been used to improve RV dysfunction compared to anticoagulation alone [3–5]. Mechanical thrombectomy was introduced as an endovascular option to avoid thrombolytics and may be considered in patients with shock, failed thrombolysis, or bleeding risk [6].

Despite increased adoption of mechanical thrombectomy for both intermediate- and high-risk PE, data directly comparing mechanical thrombectomy to CDT with respect to clinical outcomes and adverse events have been limited. Several retrospective cohort studies, the PEERLESS randomized controlled trial, and subsequent real-world analyses have addressed this comparison [7–11]. Concurrently, the HI-PEITHO trial and others have provided new evidence for catheter-based reperfusion strategies against anticoagulation alone [12]. The primary objective of this study was to utilize the Nationwide Readmissions Database (NRD) to compare CDT and mechanical thrombectomy for acute high- and intermediate-risk PE with respect to unplanned 6-month readmissions and outcomes.

## Methods

### Institutional review board statement

Institutional review board approval was not required for this study because it only contains deidentified patient data from a national public database.

### Data source

The NRD database records about 35 million discharges annually, encompassing all types of payers and uninsured patients, from over 2000 hospitals across 30 states. It analyzes readmission trends with both clinical and nonclinical factors, accounting for approximately 60% of all hospitalizations in the USA. Moreover, the NRD includes sampling weights that allow for the estimation of national rates. Further information about the NRD can be found at https://www.hcup-us.ahrq.gov/db/nation/nrd/nrddbdocumentation.jsp [13].

### Study cohort

All adult patients with a diagnosis of acute, nonseptic PE who underwent CDT or thrombectomy were identified using International Classification of Diseases, 10th Edition (ICD-10), codes. Admissions involving septic PEs were excluded using corresponding ICD codes. Additionally, patients with acute ST-elevation myocardial infarction and ischemic stroke were excluded to limit the misclassification of indication for thrombolytic use in addition to pre-procedural intracranial hemorrhage (ICH) and hemorrhagic conversion. ICD codes for shock, vasopressor usage and mechanical ventilation as well as saddle PE were included to distinguish between high risk PE from low risk which was excluded. This classification was described in previous studies [14–17]. The final cohort was split into CDT and mechanical thrombectomy (MT) based on the ICD-10 procedural codes. Patients that received intravenous thrombolytics (IVT) in combination of these procedures were excluded from our cohort. Patients with procedure codes for both CDT and MT during the index admission were excluded to maintain mutually exclusive treatment groups (*n* = 790 unweighted; weighted national estimate 1436). All codes are listed in Supplementary Table 1.

### Covariates of interest

Several demographic variables, such as age, sex, insurance status, and quartile of median household income, were gathered, alongside information on hospital characteristics such as teaching status and size. Using corresponding ICD codes, Patients with an inferior vena cava (IVC) filter were also identified. Hemodynamic shock, mechanical ventilation, and vasopressor use were used to classify high-risk PE based on prior definitions [14–16]. To assess the risks of unfavorable outcomes, the Elixhauser comorbidity indices were utilized, which consider 31 predefined comorbidities and make use of ICD-9 and ICD-10 codes [18]. In addition, the length of stay, days until readmission, and total cost for the index admission (in US dollars adjusted for inflation in 2024) were among the other variables collected. Hospital charges were converted to cost estimated using NRD-provided cost-to-charge ratios and standardized to 2024 U.S. dollars using inflation adjustment.

### Outcomes of interest

*Three* variables were collected based on diagnosis at discharge during the index admission and readmissions using the ICD codes: intracranial hemorrhage (ICH), gastrointestinal bleeding (GIB), and blood transfusion (BT) and the presence of recurrent PE. Discharges other than home (DOTH), including discharges to short-term care, skilled nursing facility, and home health care, were also captured for the index admissions. Cardiovascular-related unplanned readmissions were derived by selecting readmissions that were nonelective. Of note, readmission outcomes do not include the outcomes from the index admissions.

### Statistical analysis

The cohort was divided into CDT and MT groups. Categorical variables were compared using *χ*^2^ or Fisher exact tests, and continuous variables using t tests. Multivariable hierarchical regression adjusted for demographics, hospital characteristics, and IVC filter status was performed to evaluate associations between treatment modality and index outcomes (in-hospital mortality, DOTH, GIB, ICH, BT). Negative binomial regression was used for cost analysis. 7-day, 1-month and 6-month outcomes included unplanned readmission, GIB, ICH, BT, recurrent PE, and mortality, reported as odds ratios (ORs) with 95% confidence intervals (CIs).

Inverse probability weighted regression adjustment (IPWRA) was the primary analytic approach, estimating average treatment effects (ATE). Treatment and outcome models were adjusted for age, sex, insurance, income quartile, massive PE, IVC filter, and Elixhauser Comorbidity Index. Covariate balance before and after weighting was assessed using standardized mean differences (SMDs) and variance ratios. Adequate balance was defined as SMD < 0.1 and variance ratios near 1. This method reduces bias from measured confounders in observational studies [19]. Covariates were selected based on clinical relevance, prior literature, and univariate associations. Variables were removed only in cases of significant multicollinearity or model instability [17, 20]. Subgroup analysis for intermediate-risk PEs was completed for index admission and 6-month readmission using IPWRA. The primary analytic approach was IPWRA estimating average treatment effects (ATE), while the multivariable regression analysis was secondary. To aid interpretation, adjusted odds ratios with 95% confidence intervals were derived from the IPWRA potential-outcome means for each binary outcome and are reported alongside the average treatment effects in Table 2. All analyses were conducted using population weights provided by the NRD with “DISCWT” variable. *p* values of < 0.05 were considered statistically significant. Statistical analysis was performed using STATA 17 (StataCorp, College Station, Texas).

## Results

### Patient characteristics

A total of 28,376 patients were identified; 18,168 (64%) underwent CDT and 10,208 (36%) underwent MT. There was a total of 3945 patients with high risk PE (13.9%) (Table 1). After inverse probability weighting, covariates were well balanced (SMD < 0.10; Supplementary Table 2).

**Table 1:** Patient demographics, in *n* (%) or mean (SD)

Variables	Treatment type			
	CDT 18,168 (64.0%)	Thrombectomy alone 10,208 (36.0%)	Total 28,376 (100.0%)	*p*-value
**Demographics**				
Age in years at admission	60.01 (15.19)	61.40 (14.95)	60.51 (15.12)	< 0.001
Number of readmissions	0.22 (0.60)	0.24 (0.61)	0.23 (0.60)	0.104
Final follow-up time, in days	16.76 (34.69)	17.83 (35.90)	17.15 (35.13)	0.093
Gender				0.165
Male	9532 (52.47%)	5492 (53.80%)	15,024 (52.95%)	
Female	8636 (47.53%)	4716 (46.20%)	13,352 (47.05%)	
Primary expected payer				0.115
Medicare	8100 (44.66%)	4813 (47.23%)	12,913 (45.58%)	
Medicare	1698 (9.36%)	963 (9.45%)	2660 (9.39%)	
Private insurance	6849 (37.76%)	3605 (35.38%)	10,454 (36.90%)	
Self-pay	713 (3.93%)	412 (4.05%)	1126 (3.97%)	
No charge	105 (0.58%)	48 (0.47%)	153 (0.54%)	
Other	673 (3.71%)	349 (3.43%)	1023 (3.61%)	
Bed size of hospital				0.027
Small	2387 (13.14%)	1181 (11.57%)	3568 (12.57%)	
Medium	4965 (27.33%)	2481 (24.31%)	7446 (26.24%)	
Large	10,816 (59.54%)	6545 (64.12%)	17,362 (61.19%)	
Teaching status of urban hospitals				0.13
Urban Nonteaching	3460 (19.05%)	1584 (15.51%)	5044 (17.78%)	
Urban Teaching	13,752 (75.69%)	8188 (80.22%)	21,940 (77.32%)	
Rural	956 (5.26%)	436 (4.27%)	1392 (4.90%)	
Median household income national quartile for patient ZIP Code				0.053
0–25 percentile	5194 (28.87%)	3183 (31.52%)	8377 (29.82%)	
26–50 percentile	5070 (28.18%)	2913 (28.85%)	7983 (28.42%)	
51–75 percentile	4556 (25.32%)	2347 (23.24%)	6903 (24.58%)	
> 75 percentile	3172 (17.63%)	1655 (16.39%)	4827 (17.18%)	
IVC filter	2572 (14.16%)	1486 (14.55%)	4058 (14.30%)	0.683
Massive PE	2232 (12.29%)	1713 (16.79%)	3945 (13.90%)	< 0.001
Mechanical ventilation	1614 (8.88%)	1139 (11.16%)	2753 (9.70%)	< 0.001
Vasopressor	226 (1.25%)	236 (2.31%)	462 (1.63%)	< 0.001
Non-septic shock	1060 (5.84%)	967 (9.47%)	2027 (7.14%)	< 0.001
**Elixhauser comorbidities**				
Elixhauser Scaled Score (AHRQ Algorithm, Readmission)	19.15 (14.79)	20.74 (15.67)	19.72 (15.13)	< 0.001
Congestive heart failure	3145 (17.31%)	1877 (18.38%)	5021 (17.70%)	0.140
Cardiac arrhythmias	4472 (24.61%)	2583 (25.30%)	7054 (24.86%)	0.394
Valvular disease	1419 (7.81%)	681 (6.67%)	2099 (7.40%)	0.028
Peripheral vascular disorders	1108 (6.10%)	503 (4.93%)	1611 (5.68%)	0.004
Paralysis	130 (0.72%)	169 (1.66%)	300 (1.06%)	< 0.001
Other neurological disorders	1257 (6.92%)	969 (9.50%)	2227 (7.85%)	< 0.001
Chronic pulmonary disease	3699 (20.36%)	1880 (18.42%)	5579 (19.66%)	0.015
Diabetes, uncomplicated	2456 (13.52%)	1338 (13.11%)	3794 (13.37%)	0.518
Diabetes, complicated	2702 (14.87%)	1542 (15.10%)	4243 (14.95%)	0.742
Hypothyroidism	2180 (12.00%)	1211 (11.86%)	3391 (11.95%)	0.829
Renal failure	1990 (10.95%)	1145 (11.22%)	3135 (11.05%)	0.690
Liver disease	865 (4.76%)	589 (5.77%)	1454 (5.12%)	0.010
Peptic ulcer disease excluding bleeding	52 (0.29%)	54 (0.53%)	107 (0.38%)	0.032
AIDS/HIV	38 (0.21%)	19 (0.18%)	57 (0.20%)	0.750
Lymphoma	122 (0.67%)	95 (0.93%)	217 (0.77%)	0.100
Metastatic cancer	460 (2.53%)	449 (4.40%)	909 (3.20%)	< 0.001
Solid tumor without metastasis	890 (4.90%)	850 (8.33%)	1741 (6.13%)	< 0.001
Rheumatoid arthritis/collagen vascular	564 (3.10%)	291 (2.85%)	855 (3.01%)	0.394
Coagulopathy	3175 (17.48%)	1634 (16.01%)	4809 (16.95%)	0.039
Obesity	7595 (41.81%)	4050 (39.67%)	11,645 (41.04%)	0.035
Weight loss	597 (3.28%)	460 (4.51%)	1057 (3.72%)	< 0.001
Fluid and electrolyte disorders	5340 (29.39%)	3327 (32.59%)	8667 (30.54%)	< 0.001
Blood loss anemia	194 (1.07%)	107 (1.05%)	301 (1.06%)	0.927
Deficiency anemia	871 (4.79%)	583 (5.71%)	1454 (5.12%)	0.019
Alcohol abuse	783 (4.31%)	345 (3.38%)	1128 (3.97%)	0.008
Drug abuse	582 (3.21%)	311 (3.04%)	893 (3.15%)	0.610
Psychoses	189 (1.04%)	99 (0.97%)	288 (1.01%)	0.695
Depression	2268 (12.48%)	1298 (12.72%)	3567 (12.57%)	0.708
**Index hospitalization outcomes**				
Length of stay, in days	5.88 (6.42)	6.39 (8.73)	6.06 (7.34)	0.001
Total charge (in 2024 dollars)	34,787.89 (27,233)	41,884.06 (39,936.06)	37,339.52 (32,556.97)	< 0.001
Abdominal bleeding	346 (1.91%)	258 (2.53%)	605 (2.13%)	0.015
Intracranial hemorrhage	89 (0.49%)	121 (1.19%)	210 (0.74%)	< 0.001
Discharge type				
Routine	13,158 (75.40%)	6836 (70.43%)	19,994 (73.62%)	< 0.001
Short-term hospital	113 (0.65%)	63 (0.65%)	176 (0.65%)	
Skilled nursing/intermediate care facility	1700 (9.74%)	1165 (12.00%)	2865 (10.55%)	
Home health care (HHC)	2480 (14.21%)	1643 (16.93%)	4123 (15.18%)	
Discharge other than home	4293 (24.60%)	2871 (29.57%)	7164 (26.38%)	< 0.001
Died during hospitalization	635 (3.50%)	464 (4.54%)	1,099 (3.87%)	0.005
**1-week readmission outcomes**				
Unplanned readmission	502 (2.86%)	258 (2.65%)	760 (2.79%)	0.487
PE	245 (1.40%)	157 (1.61%)	402 (1.47%)	0.318
ICH	16 (0.09%)	19 (0.19%)	35 (0.13%)	0.195
GI bleed	18 (0.10%)	12 (0.12%)	30 (0.11%)	0.793
Blood transfusion	24 (0.14%)	23 (0.23%)	47 (0.17%)	0.209
Death	502 (2.76%)	335 (3.29%)	838 (2.95%)	0.107
**1-month readmission outcomes**				
Unplanned readmission	1,094 (6.24%)	698 (7.16%)	1,792 (6.57%)	0.058
PE	452 (2.58%)	288 (2.95%)	740 (2.71%)	0.227
ICH	32 (0.19%)	27 (0.28%)	60 (0.22%)	0.301
GI bleed	47 (0.27%)	36 (0.37%)	83 (0.30%)	0.274
Blood transfusion	44 (0.25%)	79 (0.81%)	122 (0.45%)	< 0.001
Death	675 (3.72%)	498 (4.88%)	1173 (4.13%)	0.002
**6-month outcomes**				
Unplanned readmission	2453 (14.00%)	1544 (15.85%)	3997 (14.66%)	0.006
All-cause readmission	2791 (15.92%)	1673 (17.17%)	4463 (16.37%)	0.073
Pulmonary embolism	739 (4.21%)	455 (4.67%)	1194 (4.38%)	0.238
ICH	47 (0.27%)	48 (0.49%)	95 (0.35%)	0.049
GI bleed	115 (0.66%)	110 (1.13%)	225 (0.83%)	0.004
Death at final follow-up	737 (4.06%)	546 (5.35%)	1283 (4.52%)	< 0.001

### Multivariable regression

At index admission, MT was associated with higher odds of DOTH (OR 1.15; 95% CI 1.04–1.27; *p* = 0.006), ICH (OR 1.84; 95% CI 1.20–2.83; *p* = 0.005), and BT (OR 1.51; 95% CI 1.24–1.82; *p* < 0.001). MT was also associated with 13% higher cost (Coef. 0.12; 95% CI 0.09–0.15; *p* < 0.001) (Supplementary Table 3).

At 6 months, MT was associated with higher odds of GIB (OR 1.55; 95% CI 1.06–2.28; *p* = 0.025) and BT (OR 1.46; 95% CI 1.01–2.10; *p* = 0.041). No significant differences were observed for mortality (OR 1.07; 95% CI 0.88–1.29; *p* = 0.52), ICH (OR 1.70; 95% CI 0.93–3.11; *p* = 0.082), recurrent PE (OR 1.10; 95% CI 0.92–1.33; *p* = 0.29), or unplanned readmission (OR 1.08; 95% CI 0.97–1.20; *p* = 0.18) (Supplementary Table 4).

### IPWRA treatment effects (primary analysis)

At index admission, MT was associated with higher average treatment effects (ATEs) for in-hospital mortality (0.007; *p* = 0.048), DOTH (0.031; *p* < 0.001), ICH (0.006; *p* < 0.001), BT (0.017; *p* < 0.001), and cost (0.118; *p* < 0.001), with similar GIB (0.004; *p* = 0.097).

At 30 days, MT was associated with higher mortality (ATE 0.008; *p* = 0.036) and BT (0.005; *p* < 0.001), while other outcomes were similar.

**Fig. 1:**
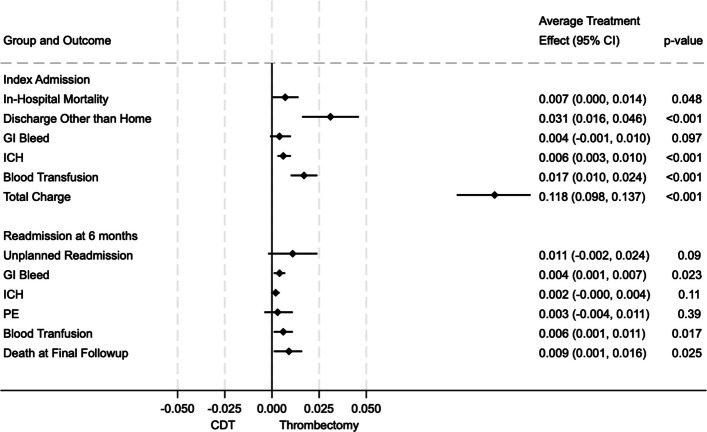
Inverse propensity weighting with regression adjustment analysis for outcomes at index admission and 6-month readmissions depicted as forest plot

At 6 months, MT was associated with small absolute increases in mortality (ATE 0.009; *p* = 0.025), GIB (0.004; *p* = 0.023), and BT (0.006; *p* = 0.017), with no significant differences in unplanned readmission (0.011; *p* = 0.09), ICH (0.002; *p* = 0.11), or recurrent PE (0.003; *p* = 0.39) (Table 2; Fig. 1).

**Table 2:** Adjusted odds ratios and average treatment effects of mechanical thrombectomy compared with CDT for outcomes in the index admission and readmission at 7 days, 30 days, and 6 months

Outcome	Adjusted OR (95% CI)	Average treatment effect	95% lower bound	95% upper bound	*p*-value
**Index admission**					
In-hospital mortality	1.20 (1.00–1.41)	0.007	0.0001	0.014	0.048
Discharge other than home	1.17 (1.09–1.26)	0.031	0.016	0.046	< 0.001
GI bleed	1.21 (0.95–1.52)	0.004	− 0.001	0.010	0.097
ICH	2.20 (1.60–3.00)	0.006	0.003	0.010	< 0.001
Blood transfusion	1.55 (1.32–1.78)	0.017	0.010	0.024	< 0.001
Total cost^1^	Mean $34,788 (CDT) vs. $41,884 (MT); median $28,827 vs. $32,615	0.118	0.098	0.137	< 0.001
**7-day readmission**					
Unplanned readmission	0.90 (0.68–1.11)	− 0.003	− 0.009	0.003	0.355
GI bleed	1.09 (0.09–2.09)^2^	0.002	− 0.002	0.007	0.354
ICH	1.90 (0.20–3.61)^2^	0.001	− 0.001	0.003	0.293
PE	1.16 (0.83–1.49)	0.000	− 0.001	0.001	0.871
Blood transfusion	1.57 (0.50–2.65)^2^	0.001	− 0.001	0.002	0.285
Death	1.13 (0.90–1.36)	− 0.003	− 0.009	0.003	0.355
**30-day readmission**					
Unplanned readmission	1.10 (0.95–1.26)	0.006	− 0.003	0.015	0.21
GI bleed	1.25 (0.61–1.93)	0.003	− 0.003	0.009	0.32
ICH	1.42 (0.42–2.43)^2^	0.001	− 0.001	0.003	0.42
PE	1.12 (0.88–1.37)	0.001	− 0.001	0.003	0.44
Blood transfusion	2.98 (1.93–4.03)	0.005	0.002	0.008	< 0.001
Death	1.21 (1.01–1.41)	0.008	0.001	0.015	0.036
**6-month readmission**					
Unplanned readmission	1.09 (0.98–1.20)	0.011	− 0.002	0.024	0.09
GI bleed	1.59 (1.15–2.04)	0.004	0.001	0.007	0.023
ICH	1.72 (0.86–2.44)	0.002	− 0.0004	0.004	0.11
PE	1.07 (0.90–1.27)	0.003	− 0.004	0.011	0.39
Blood transfusion	1.53 (1.09–1.97)	0.006	0.001	0.011	0.017
Death at final follow-up	1.23 (1.02–1.40)	0.009	0.001	0.016	0.025

Adjusted odds ratios were derived from the inverse-probability-weighted regression adjustment (IPWRA) potential-outcome means for mechanical thrombectomy versus CDT, with the corresponding average treatment effect (risk difference) shown alongside

^1^Total cost is reported as survey-weighted absolute mean and median values; the average treatment effect is on the natural-log cost scale, corresponding to approximately 12.5% higher cost with mechanical thrombectomy. Odds ratios are not reported for total cost (continuous). Readmission-linked outcomes were evaluated in 14,104 records with valid readmission linkage; in-hospital mortality reflects the full cohort (14,687)

^2^Odds ratio based on fewer than 30 events; the estimate and confidence interval are imprecise and should be interpreted with caution

The mean total hospitalization cost was $41,884 (SD $39,932) for MT compared with $34,788 (SD $27,232) for CDT (median $32,615 vs. $28,827). In IPWRA analysis, MT was associated with a 0.118 higher ln-transformed total cost (*p* < 0.001), corresponding to approximately 12.5% higher cost. Survey-weighted model-adjusted mean costs were $41,215 for MT versus $35,032 for CDT. Mean length of stay was 6.4 days for MT versus 5.9 days for CDT (*p* = 0.001).

At the first unplanned readmission within 6 months, circulatory diagnoses were most common in both groups (31.0% CDT vs. 31.6% MT). Sepsis (A419) and unspecified PE (I2699) were among the leading primary diagnoses in both cohorts, with sepsis more frequent in the MT group (7.9% vs. 5.7%) (Supplementary Table 6).

### Subgroup analysis—intermediate-risk PE

MT was associated with higher index ICH (ATE 0.004; *p* = 0.003), BT (0.012; *p* < 0.001), and cost (0.086; *p* < 0.001), with similar mortality and DOTH.

At 6 months, MT was associated with higher unplanned readmission (0.014; *p* = 0.044), GIB (0.004; *p* = 0.027), recurrent PE (0.010; *p* = 0.020), BT (0.005; *p* = 0.039), and mortality (0.005; *p* = 0.045), with similar ICH (0.001; *p* = 0.245) (Supplementary Table 5).

### Subgroup analysis—high-risk PE

In the high-risk PE subgroup (*n* = 3945 weighted), MT was associated with higher rates of discharge other than home (ATE 0.101, *p* < 0.001) and higher ln-transformed total cost (ATE 0.222, *p* < 0.001), while CDT was associated with higher 6-month PE readmission (ATE − 0.037, *p* = 0.002); in-hospital mortality did not differ significantly (ATE − 0.025, *p* = 0.19) (Supplementary Table 7). In this subgroup, the absence of a significant mortality difference and the higher PE readmission rate after CDT suggest that treatment-associated patterns may differ by PE severity.

## Discussion

The present study queried a national database to compare the risks of index hospitalization complications and 7-day, 30-day, and 6-month readmissions between CDT and MT for intermediate- and high-risk PEs. MT was associated with higher rates of DOTH, ICH, BT, and total cost at index admission, as well as a small absolute increase in 1-month and 6-month mortality in IPWRA analysis. In the subgroup analysis of intermediate-risk PE, MT demonstrated higher ATEs for ICH, BT, and total cost at index admission, and for unplanned readmission, GIB, recurrent PE, BT, and mortality at 6 months. Although inverse probability weighting improved covariate balance, residual confounding by indication remains likely, as patients undergoing mechanical thrombectomy had higher rates of massive PE and hemodynamic instability at baseline. While IPWRA demonstrated a small absolute increase in 6-month mortality, this association was not observed in multivariable regression, underscoring sensitivity to modeling approach and the potential influence of residual confounding. Long-term outcomes likely reflect cumulative patient trajectory, post-discharge anticoagulation management, and residual confounding rather than procedural effects alone.

Prospective device studies have reported favorable short-term safety in selected populations, including low major bleeding in the FLARE trial evaluating the FlowTriever system and reduced ICH rates in the SEATTLE II trial evaluating ultrasound-assisted CDT [21, 22]. Similarly, short-term thrombectomy outcomes through 30 days were reported in the EXTRACT-PE trial evaluating the Indigo Aspiration System [23]. In contrast, our real-world NRD analysis demonstrated higher adjusted ICH and BT at index admission with MT and higher 6-month GIB, underscoring differences between trial-selected populations and routine clinical practice [21–23]. Furthermore, the findings suggest that MT was associated with higher rates of DOTH, BT, ICH, and total cost at index admission, and a small absolute increase in mortality at 1- and 6-month follow-up in IPWRA analysis, while demonstrating similar rates of unplanned readmission at early time points and higher GIB and unplanned readmission at 6 months.

The higher rates of ICH, blood transfusion, and GI bleeding in the MT cohort merit discussion of alternative explanations. Residual confounding may be important, as patients selected for MT had greater baseline illness severity and a higher cancer burden, both of which carry independent bleeding risk. ICD-10 coding misclassification is a relevant consideration, as procedure-related blood loss from large-bore vascular access may be variably coded across institutions. Variation in periprocedural anticoagulation practices, which cannot be captured in administrative data, may also contribute. Finally, MT is often selected for patients considered unsuitable for thrombolysis due to elevated bleeding risk, which may concentrate bleeding-prone patients in the MT cohort. These findings are consistent with REAL-PE and REAL-PE II. As with all observational analyses, causality cannot be inferred from these data alone.

The PEERLESS randomized controlled trial compared large-bore MT directly to CDT in intermediate-risk PE and reported favorable outcomes for MT using a hierarchical composite win ratio endpoint [7]. The composite included both softer measures such as ICU admission duration, need for rescue therapy, and early clinical deterioration, alongside harder endpoints like mortality and intracranial hemorrhage. Notably, when individual components were examined, no significant differences were identified between groups for mortality or ICH [7]. This distinction matters: win ratio composites can convey an impression of broad superiority that does not always translate into patient-centered benefit when softer, resource-based outcomes account for much of the signal. PEERLESS also enrolled hemodynamically stable intermediate-risk patients under controlled trial conditions, likely excluding those with the highest baseline risk profile where procedural safety margins are narrowest. Our results, alongside REAL-PE and other real-world observational analyses, suggest that outcomes observed in trial populations may not fully generalize to the broader spectrum of patients receiving MT in routine clinical practice [9, 11, 25–27].

The recently published HI-PEITHO trial offers additional context for evaluating catheter-based reperfusion strategies in intermediate-high-risk PE. In this multicenter randomized trial, ultrasound-facilitated CDT reduced the composite outcome of hemodynamic decompensation or death at 7 days by approximately 61% compared with anticoagulation alone [12]. Rates of major bleeding and intracranial hemorrhage were not significantly different between groups, supporting the acceptable safety profile of catheter-directed thrombolytics at reduced doses. These findings reinforce the continued therapeutic value of CDT and raise reasonable questions about whether the added procedural complexity and cost of large-bore mechanical thrombectomy are justified across all intermediate-risk PE presentations, particularly in hemodynamically stable patients. Additionally, our subgroup findings should be interpreted cautiously given ICD-based risk classification and multiple comparisons.

The etiology of higher DOTH after MT is uncertain and may reflect unmeasured severity or real-world practice variation [28]. MT was associated with higher DOTH (ATE 0.031; *p* < 0.001) and higher BT/ICH, which could relate to operator experience, device learning curves, or incomplete thrombus extraction, particularly early in adoption [21]. Although IPWRA reduces bias from measured confounders, residual unmeasured confounding likely persists [28]. While large retrospective database analyses provide valuable real-world insights, future studies in this field should ideally be prospective in design to better address confounding and causal inference.

The economic implications of device selection deserve careful consideration in the context of PE care. In the present study, MT was associated with significantly higher total hospitalization costs compared with CDT, with an average treatment effect of 0.118 (*p* < 0.001) in IPWRA analysis. This cost differential persisted in the intermediate-risk PE subgroup (ATE 0.086; *p* < 0.001), where procedural urgency is often lower and patient trajectories are generally favorable with anticoagulation or reduced-intensity reperfusion alone. These findings are consistent with prior cost-comparison analyses demonstrating substantially higher expenditures for large-bore thrombectomy systems relative to catheter-directed thrombolytic delivery [10]. Large-bore mechanical thrombectomy devices carry per-case costs that can exceed $8000 to $12,000, while catheter-directed administration of recombinant tissue plasminogen activator uses generically available pharmaceuticals at a fraction of that cost [29]. The total cost differential likely also reflects downstream effects, including higher rates of mechanical ventilation (11.2% vs. 8.9%; *p* = 0.005), periprocedural complications, and longer hospital stays (mean 6.4 vs. 5.9 days; *p* = 0.001). Captured across more than 28,000 procedures representing approximately 60% of U.S. hospitalizations, the aggregate economic burden of routine MT adoption in intermediate-risk patients without hemodynamic compromise could be substantial at a national level. Prospective analyses incorporating quality-adjusted outcomes and long-term resource utilization will be important for guiding rational device selection across PE risk strata.

### Strengths and limitations

Although this study was a comprehensive national-level analysis, there are several limitations. Administrative claims data was utilized to identify diagnoses and procedures, which is prone to human error and misclassification of the ICD codes [30]. Specifically, the stratification of risk of PE in this current study was based on ICD codes. However, codes selected for this study were curated by clinicians and billing code experts as well as industry billing guides [17, 29, 31, 32]. Moreover, heparin use and dosage rates were not able to be recorded due to lack of corresponding ICD codes. Those who underwent IVT were excluded, which limits the analysis to admission that did not require IVT either before or after the studied intervention. The NRD does not capture in-hospital fibrinolytic dosing, and some patients undergoing MT may have received adjunctive thrombolytics, potentially influencing bleeding outcomes. Claims-based data cannot distinguish procedural complications from bleeding related to anticoagulation or comorbidities. Additionally, there are less than 11 patients with brain tumor, and among them with ICH. These patients are unlikely to meaningfully influence overall results. Due to the nature of retrospective data, there may be selection bias with procedural choices based on risk factors and that patients who received MT may have required faster intervention than the slower thrombolytic infusion of CDT. As with other claims-based analyses, individual thrombectomy devices and CDT delivery systems cannot be distinguished. The MT cohort likely encompasses multiple large-bore aspiration, rheolytic, and combined thrombectomy platforms with differing technical profiles. The CDT cohort may similarly include both standard infusion catheters and ultrasound-facilitated delivery systems. Thrombectomy technology evolved across the 2016 to 2022 study period, and these results reflect the composite real-world experience during this period rather than any specific device or technique. Although IPWRA reduced bias from measured confounders, unmeasured clinical severity variables and selection factors may still influence outcomes. Additionally, although we utilized the ECI score for risk stratification, the database does not capture PE-specific physiologic severity markers such as RV dysfunction, clot burden, laboratory values, or operator decision-making factors. Lastly, cause of mortality is not recorded. Mortality in this study reflects in-hospital death during index admission or subsequent readmissions within 7, 30, and 180 days, and does not capture out-of-hospital mortality.

As with all observational database studies, residual confounding by indication cannot be excluded. IPWRA achieved standardized mean differences below 0.10 for all measured covariates; however, key clinical parameters that influence treatment selection, including RV/LV ratio, clot burden, troponin, BNP, and oxygen requirement, are not available in administrative claims data. Patients undergoing MT had higher baseline rates of massive PE, vasopressor use, and mechanical ventilation, and these baseline differences should be considered when interpreting the observed associations. Real-world database analyses nonetheless provide important evidence across the broad spectrum of patients encountered in clinical practice, complementing findings from randomized trials conducted in more selected populations [24].

The classification of PE severity using administrative ICD-10 codes does not fully replicate contemporary guideline-based risk stratification. Key clinical parameters including RV/LV ratio, troponin, BNP, echocardiographic findings, and oxygen requirements are not available in the NRD. High-risk PE was approximated using markers of hemodynamic compromise consistent with prior database studies; however, intermediate-risk patients in this cohort may represent a heterogeneous population that would not uniformly satisfy contemporary definitions of intermediate-high-risk PE. This is a known constraint of administrative database research in this field.

## Conclusion

In this national analysis, mechanical thrombectomy was associated with higher rates of adverse in-hospital events and total cost compared with catheter-directed thrombolysis, as well as a small absolute increase in 6-month mortality on primary analysis. These findings reflect real-world patterns across a nationally representative cohort and should be considered alongside the observational study design. Prospective randomized data are needed to define the optimal role of each strategy in contemporary PE care.

## Supplementary Information

Supplementary Material 1: Supplementary Table 1: ICD codes used in this study. Supplementary Table 2: Covariate balance before and after weighting. Supplementary Table 3: Multivariable Regression for mortality, DOTH, GI bleed, ICH, blood transfusion and total cost at index admission. Supplementary Table 4: Multivariable Regression for unplanned readmission, GI bleed, ICH, recurrent pulmonary embolism, blood transfusion, and mortality at 6-month follow-up. Supplementary Table 5: Average treatment effect of Thrombectomy alone compared to CDT for outcomes in the index admission and admission at 6 months for intermediate-risk PEs. Supplementary Table 6. Primary Diagnoses at First Unplanned Readmission Within 6 Months, by Treatment Group. Supplementary Table 7. Inverse Probability Weighted Regression Adjustment (IPWRA) Results — High-Risk Pulmonary Embolism Subgroup.

## Data Availability

The data that support the findings of this study are available from the Healthcare Cost and Utilization Project (HCUP) Nationwide Readmissions Database (NRD). Data are available for purchase at https://www.hcup-us.ahrq.gov/db/nation/nrd/nrddbdocumentation.jsp.

## Abbreviations

ATE: Average treatment effect
BT: Blood transfusion
CDT: Catheter-directed thrombolysis
DOTH: Discharge other than home
GIB: Gastrointestinal bleeding
ICH: Intracranial hemorrhage
MT: Mechanical thrombectomy
PE: Pulmonary embolism
NRD: Nationwide Readmissions Database
OR: Odds ratio
IPWRA: Inverse propensity weighted regression adjustment
CI: Confidence interval
