# Supplementary material for: Unplanned 6-month readmissions and in-hospital outcomes for the management of acute intermediate- and high-risk pulmonary embolism: comparisons of endovascular interventions
Source: CVIR Endovasc. 2026 Aug 12;9:99. doi: 10.1186/s42155-026-00752-1 (PMC13469043; doi:10.1186/s42155-026-00752-1)
Supplement: Supplementary file 1 [file 42155_2026_752_MOESM1_ESM.docx]

Supplementary Table 1: ICD codes used in this study

| Variables | ICD-10 |
| --- | --- |
| Pulmonary Embolism | I26.02, I26.92, I26.99 |
| Septic PE (Excluded) | I26.90, I26.01 |
| History of PE (Excluded) | Z86.11 |
| Massive PE includes Code for PE + any of three below |  |
| Mechanical Ventilation | 5A1945Z, 5A1955Z, 5A1935Z, 5A09357, 5A09457, 5A09557 |
|  | Vent dependent: Z99.11 |
| Vasopressor Use | 3E030XZ, 3E033XZ, 3E040XZ, 3E043XZ, 3E050XZ, 3E053XZ, 3E060XZ, 3E063XZ |
| Non-Septic Shock | R57 |
|  | Exclude septic: R65.21 |
| IVC Filter | 06H03DZ |
| Intracerebral Hemorrhage | I60, I61, I62 |
| Abdominal bleeding | I6781, I6782, I8501, I8511, K2211, K250, K252, K254, K256, K260, K262, K264, K266, K270, K272, K274, K276, K280, K282, K284, K286, K2901, K2921, K2931, K2941, K2951, K2961, K2971, K2981, K2991, K31811, K920, K922, K661, K5521, K921, K625 |
| Blood Transfusions | 30230H0, 30230H1, 30233H, 30233H0, 30233H1, 30240H, 30240H0, 30240H1, 30243H, 30243H0, 30243H1, 30230N, 30230N0, 30230N1, 30233N, 30233N0, 30233N1, 30240N, 30240N0, 30240N1, 30243N, 30243N0, 30243N1 |
| Thrombolytic Therapy (Endo) | Thrombolysis (excluded): 3E03317 |
|  | CDT: 3E06317 |
| Thrombectomy | 02CP3ZZ, 02CQ3ZZ, 02CR3ZZ, 02CS3ZZ, 02CT3ZZ |
| Embolectomy (Surgery), Excluded | 02CP0ZZ, 02CQ0ZZ, 02CQ4ZZ, 02CR0ZZ, |
|  | 02CP4ZZ, 02CR4ZZ |

**Supplementary Table 2**: Covariate balance before and after weighting

| **Variable** | **Std Diff (Raw)** | **Std Diff (Weighted)** | **Variance Ratio (Raw)** | **Variance Ratio (Weighted)** |
| --- | --- | --- | --- | --- |
| **Age** | 0.0983 | 0.0003 | 0.9632 | 1.0112 |
| **Female (vs Male)** | -0.0269 | 0.0002 | 0.9965 | 1.0000 |
| **Insurance Status (vs Medicare)** |  |  |  |  |
| Medicaid | -0.0022 | 0.0011 | 0.9941 | 1.0031 |
| Private insurance | -0.0548 | 0.0001 | 0.9693 | 1.0000 |
| Self-pay | 0.0018 | 0.0004 | 1.0088 | 1.0020 |
| No charge | -0.0160 | -0.0010 | 0.8059 | 0.9872 |
| Other | -0.0190 | -0.0006 | 0.9089 | 0.9970 |
| **Household income quartile (vs 0–25 Percentile)** |  |  |  |  |
| 26–50 Percentile | 0.0063 | 0.0013 | 1.0065 | 1.0013 |
| 51–75 Percentile | -0.0422 | -0.0011 | 0.9520 | 0.9988 |
| >75 Percentile | -0.0277 | -0.0001 | 0.9569 | 0.9999 |
| **Massive PE (vs Submassive)** | 0.1254 | 0.0003 | 1.3404 | 1.0007 |
| **Presence of IVC Filter** | 0.0237 | -0.0029 | 1.0482 | 0.9943 |
| **Elixhauser Comorbidity Index** | 0.1018 | 0.0006 | 1.1412 | 1.0222 |

**Supplementary Table 3:** Multivariable Regression for mortality, DOTH, GI bleed, ICH, blood transfusion and total cost at index admission

| **Died during hospitalization** | | | | |
| --- | --- | --- | --- | --- |
|  | Odds Ratio | 95% lower bound | 95% upper bound | p-value |
| **Treatment Type** |  |  |  |  |
| **CDT** | 1.00 |  |  |  |
| **Thrombectomy Alone** | 1.01 | 0.82 | 1.25 | 0.907 |
| **Age in years at admission** | 1.01 | 1.01 | 1.02 | <0.001 |
| **Female [vs. Male]** | 1.31 | 1.08 | 1.60 | 0.007 |
| **Teaching status of urban hospitals** |  |  |  |  |
| **Urban Nonteaching** | 1.00 |  |  |  |
| **Urban Teaching** | 0.99 | 0.77 | 1.27 | 0.930 |
| **Rural** | 0.84 | 0.53 | 1.32 | 0.446 |
| **Median household income national quartile for patient ZIP Code** |  |  |  |  |
| **0-25 Percentile** | 1.00 |  |  |  |
| **26-50 Percentile** | 1.08 | 0.84 | 1.40 | 0.558 |
| **51-75 Percentile** | 0.97 | 0.73 | 1.29 | 0.823 |
| **>75 Percentile** | 0.85 | 0.62 | 1.17 | 0.322 |
| **IVC Filter** | 0.74 | 0.58 | 0.96 | 0.024 |
| **Massive PE** | 19.12 | 15.16 | 24.11 | <0.001 |
| **Elixhauser Scaled Score (AHRQ Algorithm, Readmission)** | 1.02 | 1.01 | 1.03 | <0.001 |
| **Discharge other than Home** | | | | |
|  | Odds Ratio | 95% lower bound | 95% upper bound | p-value |
| **Treatment Type** |  |  |  |  |
| **CDT** | 1.00 |  |  |  |
| **Thrombectomy Alone** | 1.15 | 1.04 | 1.27 | 0.006 |
| **Age in years at admission** | 1.04 | 1.04 | 1.05 | <0.001 |
| **Female [vs. Male]** | 1.89 | 1.72 | 2.07 | <0.001 |
| **Teaching status of urban hospitals** |  |  |  |  |
| **Urban Nonteaching** | 1.00 |  |  |  |
| **Urban Teaching** | 0.87 | 0.72 | 1.06 | 0.163 |
| **Rural** | 0.54 | 0.47 | 0.61 | <0.001 |
| **Median household income national quartile for patient ZIP Code** | 0.31 | 0.22 | 0.45 | <0.001 |
| **0-25 Percentile** | 0.23 | 0.08 | 0.67 | 0.007 |
| **26-50 Percentile** | 0.87 | 0.67 | 1.13 | 0.302 |
| **51-75 Percentile** |  |  |  |  |
| **>75 Percentile** | 1.00 |  |  |  |
| **IVC Filter** | 1.13 | 1.01 | 1.28 | 0.039 |
| **Massive PE** | 0.84 | 0.66 | 1.09 | 0.191 |
| **Primary expected payer (uniform)** |  |  |  |  |
| **Medicare** | 1.00 |  |  |  |
| **Medicaid** | 0.91 | 0.80 | 1.04 | 0.156 |
| **Private insurance** | 0.91 | 0.80 | 1.04 | 0.156 |
| **Self-pay** | 0.98 | 0.85 | 1.13 | 0.803 |
| **No charge** | 1.63 | 1.44 | 1.85 | <0.001 |
| **Other** | 3.43 | 3.01 | 3.92 | <0.001 |
| **Elixhauser Scaled Score (AHRQ Algorithm, Readmission)** | 1.03 | 1.03 | 1.03 | <0.001 |
| **GI Bleed** | | | | |
|  | Odds Ratio | 95% lower bound | 95% upper bound | p-value |
| **Treatment Type** |  |  |  |  |
| **CDT** | 1.00 |  |  |  |
| **Thrombectomy Alone** | 1.10 | 0.86 | 1.40 | 0.469 |
| **Age in years at admission** | 1.01 | 1.00 | 1.02 | 0.117 |
| **Female [vs. Male]** | 1.04 | 0.82 | 1.31 | 0.767 |
| **Teaching status of urban hospitals** |  |  |  |  |
| **Urban Nonteaching** | 1.00 |  |  |  |
| **Urban Teaching** | 0.73 | 0.43 | 1.22 | 0.225 |
| **Rural** | 0.99 | 0.69 | 1.40 | 0.937 |
| **Median household income national quartile for patient ZIP Code** | 1.35 | 0.71 | 2.57 | 0.361 |
| **0-25 Percentile** | 0.81 | 0.11 | 6.11 | 0.835 |
| **26-50 Percentile** | 1.32 | 0.75 | 2.33 | 0.342 |
| **51-75 Percentile** |  |  |  |  |
| **>75 Percentile** | 1.00 |  |  |  |
| **IVC Filter** | 1.04 | 0.77 | 1.42 | 0.786 |
| **Massive PE** | 0.77 | 0.37 | 1.64 | 0.504 |
| **Primary expected payer (uniform)** |  |  |  |  |
| **Medicare** | 1.00 |  |  |  |
| **Medicaid** | 0.99 | 0.71 | 1.39 | 0.948 |
| **Private insurance** | 1.06 | 0.76 | 1.47 | 0.721 |
| **Self-pay** | 1.03 | 0.71 | 1.50 | 0.867 |
| **No charge** | 3.61 | 2.79 | 4.67 | <0.001 |
| **Other** | 2.66 | 2.04 | 3.47 | <0.001 |
| **Elixhauser Scaled Score (AHRQ Algorithm, Readmission)** | 1.03 | 1.02 | 1.04 | <0.001 |
| **Intracranial Hemorrhage** | | | | |
|  | Odds Ratio | 95% lower bound | 95% upper bound | p-value |
| **Treatment Type** |  |  |  |  |
| **CDT** | 1.00 |  |  |  |
| **Thrombectomy Alone** | 1.84 | 1.20 | 2.83 | 0.005 |
| **Age in years at admission** | 1.00 | 0.98 | 1.02 | 0.855 |
| **Female [vs. Male]** | 0.86 | 0.57 | 1.29 | 0.458 |
| **Teaching status of urban hospitals** |  |  |  |  |
| **Urban Nonteaching** | 1.00 |  |  |  |
| **Urban Teaching** | 1.59 | 0.90 | 2.80 | 0.112 |
| **Rural** | 1.11 | 0.25 | 4.92 | 0.887 |
| **Median household income national quartile for patient ZIP Code** |  |  |  |  |
| **0-25 Percentile** | 1.00 |  |  |  |
| **26-50 Percentile** | 0.73 | 0.42 | 1.26 | 0.254 |
| **51-75 Percentile** | 0.76 | 0.43 | 1.33 | 0.338 |
| **>75 Percentile** | 1.10 | 0.59 | 2.06 | 0.768 |
| **IVC Filter** | 5.59 | 3.64 | 8.58 | <0.001 |
| **Massive PE** | 5.32 | 3.41 | 8.30 | <0.001 |
| **Elixhauser Scaled Score (AHRQ Algorithm, Readmission)** | 1.02 | 1.01 | 1.03 | <0.001 |
| **Blood Transfusion** | | | | |
|  | Odds Ratio | 95% lower bound | 95% upper bound | p-value |
| **Treatment Type** |  |  |  |  |
| **CDT** | 1.00 |  |  |  |
| **Thrombectomy Alone** | 1.51 | 1.24 | 1.82 | <0.001 |
| **Age in years at admission** | 1.01 | 1.00 | 1.02 | 0.077 |
| **Female [vs. Male]** | 2.50 | 2.05 | 3.05 | <0.001 |
| **Primary expected payer (uniform)** |  |  |  |  |
| **Medicare** | 1.00 |  |  |  |
| **Medicaid** | 1.68 | 1.20 | 2.34 | 0.002 |
| **Private insurance** | 1.20 | 0.92 | 1.58 | 0.180 |
| **Self-pay** | 2.15 | 1.34 | 3.46 | 0.002 |
| **No charge** | 0.50 | 0.07 | 3.82 | 0.506 |
| **Other** | 1.08 | 0.59 | 1.95 | 0.806 |
| **Teaching status of urban hospitals** |  |  |  |  |
| **Urban Nonteaching** | 1.00 |  |  |  |
| **Urban Teaching** | 1.17 | 0.90 | 1.51 | 0.237 |
| **Rural** | 1.33 | 0.77 | 2.32 | 0.307 |
| **Median household income national quartile for patient ZIP Code** |  |  |  |  |
| **0-25 Percentile** | 1.00 |  |  |  |
| **26-50 Percentile** | 1.10 | 0.86 | 1.41 | 0.451 |
| **51-75 Percentile** | 0.99 | 0.76 | 1.27 | 0.909 |
| **>75 Percentile** | 1.10 | 0.84 | 1.46 | 0.491 |
| **IVC Filter** | 2.48 | 2.01 | 3.07 | <0.001 |
| **Massive PE** | 2.96 | 2.41 | 3.64 | <0.001 |
| **Elixhauser Scaled Score (AHRQ Algorithm, Readmission)** | 1.03 | 1.03 | 1.04 | <0.001 |
| **Total Cost** | | | | |
|  | Coefficient | 95% lower bound | 95% upper bound | p-value |
| **Treatment Type** |  |  |  |  |
| **CDT** | 0.000 |  |  |  |
| **Thrombectomy Alone** | 0.117 | 0.088 | 0.147 | <0.001 |
| **Age in years at admission** | -0.002 | -0.003 | -0.001 | <0.001 |
| **Female [vs. Male]** | -0.029 | -0.052 | -0.006 | 0.014 |
| **Primary expected payer (uniform)** |  |  |  |  |
| **Medicare** | 0.000 |  |  |  |
| **Medicaid** | 0.137 | 0.079 | 0.195 | <0.001 |
| **Private insurance** | 0.003 | -0.029 | 0.035 | 0.839 |
| **Self-pay** | -0.005 | -0.066 | 0.057 | 0.879 |
| **No charge** | -0.088 | -0.234 | 0.058 | 0.236 |
| **Other** | -0.003 | -0.060 | 0.054 | 0.909 |
| **Teaching status of urban hospitals** |  |  |  |  |
| **Urban Nonteaching** | 0.000 |  |  |  |
| **Urban Teaching** | 0.070 | 0.038 | 0.102 | <0.001 |
| **Rural** | 0.115 | 0.054 | 0.176 | <0.001 |
| **Median household income national quartile for patient ZIP Code** |  |  |  |  |
| **0-25 Percentile** | 0.000 |  |  |  |
| **26-50 Percentile** | 0.043 | 0.009 | 0.077 | 0.013 |
| **51-75 Percentile** | 0.074 | 0.044 | 0.103 | <0.001 |
| **>75 Percentile** | 0.152 | 0.116 | 0.188 | <0.001 |
| **IVC Filter** | 0.292 | 0.257 | 0.326 | <0.001 |
| **Massive PE** | 0.484 | 0.442 | 0.525 | <0.001 |
| **Elixhauser Scaled Score (AHRQ Algorithm, Readmission)** | 0.010 | 0.009 | 0.010 | <0.001 |

**Supplementary Table 4:** Multivariable Regression for unplanned readmission, GI bleed, ICH, recurrent pulmonary embolism, blood transfusion, and mortality at 6-month follow-up

| **Unplanned Readmission** | | | | |
| --- | --- | --- | --- | --- |
|  | Odds Ratio | 95% lower bound | 95% upper bound | p-value |
| **Treatment Type** |  |  |  |  |
| **CDT** | 1.00 |  |  |  |
| **Thrombectomy Alone** | 1.08 | 0.97 | 1.20 | 0.182 |
| **Age in years at admission** | 1.00 | 1.00 | 1.01 | 0.925 |
| **Female [vs. Male]** | 1.06 | 0.96 | 1.17 | 0.267 |
| **Teaching status of urban hospitals** |  |  |  |  |
| **Urban Nonteaching** | 1.00 |  |  |  |
| **Urban Teaching** | 1.16 | 0.94 | 1.43 | 0.163 |
| **Rural** | 0.65 | 0.56 | 0.76 | <0.001 |
| **Median household income national quartile for patient ZIP Code** | 1.10 | 0.82 | 1.46 | 0.526 |
| **0-25 Percentile** | 1.02 | 0.54 | 1.91 | 0.963 |
| **26-50 Percentile** | 0.79 | 0.60 | 1.06 | 0.115 |
| **51-75 Percentile** |  |  |  |  |
| **>75 Percentile** | 1.00 |  |  |  |
| **IVC Filter** | 0.96 | 0.85 | 1.09 | 0.546 |
| **Massive PE** | 0.85 | 0.66 | 1.11 | 0.234 |
| **Primary expected payer (uniform)** |  |  |  |  |
| **Medicare** | 1.00 |  |  |  |
| **Medicaid** | 0.88 | 0.77 | 1.00 | 0.056 |
| **Private insurance** | 0.86 | 0.74 | 0.99 | 0.031 |
| **Self-pay** | 0.78 | 0.66 | 0.92 | 0.002 |
| **No charge** | 1.45 | 1.27 | 1.65 | <0.001 |
| **Other** | 1.16 | 1.00 | 1.35 | 0.052 |
| **Elixhauser Scaled Score (AHRQ Algorithm, Readmission)** | 1.03 | 1.03 | 1.04 | <0.001 |
| **GI Bleed** | | | | |
|  | Odds Ratio | 95% lower bound | 95% upper bound | p-value |
| **Treatment Type** |  |  |  |  |
| **CDT** | 1.00 |  |  |  |
| **Thrombectomy Alone** | 1.55 | 1.06 | 2.28 | 0.025 |
| **Age in years at admission** | 1.01 | 1.00 | 1.03 | 0.104 |
| **Female [vs. Male]** | 1.00 | 0.69 | 1.45 | 0.991 |
| **Teaching status of urban hospitals** |  |  |  |  |
| **Urban Nonteaching** | 1.00 |  |  |  |
| **Urban Teaching** | 1.16 | 0.68 | 1.97 | 0.598 |
| **Rural** | 1.59 | 0.63 | 3.97 | 0.323 |
| **Median household income national quartile for patient ZIP Code** |  |  |  |  |
| **0-25 Percentile** | 1.00 |  |  |  |
| **26-50 Percentile** | 0.93 | 0.58 | 1.51 | 0.776 |
| **51-75 Percentile** | 0.87 | 0.52 | 1.44 | 0.588 |
| **>75 Percentile** | 0.69 | 0.35 | 1.36 | 0.285 |
| **IVC Filter** | 1.53 | 0.96 | 2.45 | 0.074 |
| **Massive PE** | 0.77 | 0.44 | 1.35 | 0.369 |
| **Elixhauser Scaled Score (AHRQ Algorithm, Readmission)** | 1.04 | 1.03 | 1.05 | <0.001 |
| **ICH** | | | | |
|  | Odds Ratio | 95% lower bound | 95% upper bound | p-value |
| **Treatment Type** |  |  |  |  |
| **CDT** | 1.00 |  |  |  |
| **Thrombectomy Alone** | 1.70 | 0.93 | 3.11 | 0.082 |
| **Age in years at admission** | 1.00 | 0.97 | 1.03 | 0.942 |
| **Female [vs. Male]** | 0.75 | 0.41 | 1.38 | 0.354 |
| **Teaching status of urban hospitals** |  |  |  |  |
| **Urban Nonteaching** | 1.00 |  |  |  |
| **Urban Teaching** | 0.75 | 0.35 | 1.63 | 0.470 |
| **Rural** | 0.43 | 0.19 | 0.96 | 0.039 |
| **Median household income national quartile for patient ZIP Code** |  |  |  |  |
| **0-25 Percentile** | 1.00 |  |  |  |
| **26-50 Percentile** | 1.94 | 0.83 | 4.52 | 0.126 |
| **51-75 Percentile** | 1.19 | 0.48 | 2.99 | 0.706 |
| **>75 Percentile** | 1.53 | 0.60 | 3.94 | 0.376 |
| **IVC Filter** | 2.71 | 1.39 | 5.26 | 0.003 |
| **Massive PE** | 0.93 | 0.38 | 2.23 | 0.865 |
| **Elixhauser Scaled Score (AHRQ Algorithm, Readmission)** | 1.03 | 1.02 | 1.05 | <0.001 |
| **Pulmonary Embolism** | | | | |
|  | Odds Ratio | 95% lower bound | 95% upper bound | p-value |
| **Treatment Type** |  |  |  |  |
| **CDT** | 1.00 |  |  |  |
| **Thrombectomy Alone** | 1.10 | 0.92 | 1.33 | 0.285 |
| **Age in years at admission** | 0.98 | 0.97 | 0.99 | <0.001 |
| **Female [vs. Male]** | 0.94 | 0.79 | 1.12 | 0.478 |
| **Teaching status of urban hospitals** |  |  |  |  |
| **Urban Nonteaching** | 1.00 |  |  |  |
| **Urban Teaching** | 0.98 | 0.71 | 1.35 | 0.901 |
| **Rural** | 0.56 | 0.43 | 0.73 | <0.001 |
| **Median household income national quartile for patient ZIP Code** | 0.86 | 0.56 | 1.34 | 0.513 |
| **0-25 Percentile** | 0.90 | 0.34 | 2.37 | 0.825 |
| **26-50 Percentile** | 0.70 | 0.44 | 1.12 | 0.136 |
| **51-75 Percentile** |  |  |  |  |
| **>75 Percentile** | 1.00 |  |  |  |
| **IVC Filter** | 0.94 | 0.76 | 1.18 | 0.608 |
| **Massive PE** | 0.65 | 0.38 | 1.12 | 0.125 |
| **Primary expected payer (uniform)** |  |  |  |  |
| **Medicare** | 1.00 |  |  |  |
| **Medicaid** | 0.88 | 0.69 | 1.11 | 0.276 |
| **Private insurance** | 0.93 | 0.73 | 1.17 | 0.516 |
| **Self-pay** | 0.80 | 0.61 | 1.05 | 0.110 |
| **No charge** | 1.07 | 0.83 | 1.37 | 0.602 |
| **Other** | 0.93 | 0.70 | 1.23 | 0.600 |
| **Elixhauser Scaled Score (AHRQ Algorithm, Readmission)** | 1.03 | 1.02 | 1.03 | <0.001 |
| **Blood Transfusion** | | | | |
|  | Odds Ratio | 95% lower bound | 95% upper bound | p-value |
| **Treatment Type** |  |  |  |  |
| **CDT** | 1.00 |  |  |  |
| **Thrombectomy Alone** | 1.46 | 1.01 | 2.10 | 0.041 |
| **Age in years at admission** | 1.01 | 0.99 | 1.03 | 0.351 |
| **Female [vs. Male]** | 2.24 | 1.58 | 3.19 | <0.001 |
| **Teaching status of urban hospitals** |  |  |  |  |
| **Urban Nonteaching** | 1.00 |  |  |  |
| **Urban Teaching** | 1.38 | 0.70 | 2.74 | 0.351 |
| **Rural** | 1.09 | 0.61 | 1.97 | 0.764 |
| **Median household income national quartile for patient ZIP Code** | 1.59 | 0.63 | 4.06 | 0.328 |
| **0-25 Percentile** | 1.00 |  |  |  |
| **26-50 Percentile** | 1.33 | 0.47 | 3.82 | 0.590 |
| **51-75 Percentile** |  |  |  |  |
| **>75 Percentile** | 1.00 |  |  |  |
| **IVC Filter** | 0.91 | 0.58 | 1.44 | 0.688 |
| **Massive PE** | 0.47 | 0.14 | 1.63 | 0.235 |
| **Primary expected payer (uniform)** |  |  |  |  |
| **Medicare** | 1.00 |  |  |  |
| **Medicaid** | 0.81 | 0.50 | 1.31 | 0.387 |
| **Private insurance** | 0.98 | 0.59 | 1.61 | 0.922 |
| **Self-pay** | 1.23 | 0.73 | 2.06 | 0.435 |
| **No charge** | 1.76 | 1.13 | 2.74 | 0.012 |
| **Other** | 1.11 | 0.71 | 1.75 | 0.651 |
| **Elixhauser Scaled Score (AHRQ Algorithm, Readmission)** | 1.04 | 1.04 | 1.05 | <0.001 |
| **Death Final Followup** | | | | |
|  | Odds Ratio | 95% lower bound | 95% upper bound | p-value |
| **Treatment Type** |  |  |  |  |
| **CDT** | 1.00 |  |  |  |
| **Thrombectomy Alone** | 1.07 | 0.88 | 1.29 | 0.524 |
| **Age in years at admission** | 1.02 | 1.01 | 1.02 | <0.001 |
| **Female [vs. Male]** | 1.39 | 1.16 | 1.67 | <0.001 |
| **Teaching status of urban hospitals** |  |  |  |  |
| **Urban Nonteaching** | 1.00 |  |  |  |
| **Urban Teaching** | 1.01 | 0.80 | 1.28 | 0.923 |
| **Rural** | 1.07 | 0.73 | 1.59 | 0.720 |
| **Median household income national quartile for patient ZIP Code** |  |  |  |  |
| **0-25 Percentile** | 1.00 |  |  |  |
| **26-50 Percentile** | 1.14 | 0.90 | 1.45 | 0.265 |
| **51-75 Percentile** | 0.96 | 0.74 | 1.26 | 0.792 |
| **>75 Percentile** | 0.84 | 0.63 | 1.13 | 0.248 |
| **IVC Filter** | 0.77 | 0.60 | 0.97 | 0.028 |
| **Massive PE** | 12.18 | 10.03 | 14.80 | <0.001 |
| **Elixhauser Scaled Score (AHRQ Algorithm, Readmission)** | 1.03 | 1.02 | 1.03 | <0.001 |

**Supplementary Table 5**: Average treatment effect of Thrombectomy alone compared to CDT for outcomes in the index admission and admission at 6 months for intermediate-risk PEs

|  | Treatment Type | | | |
| --- | --- | --- | --- | --- |
|  | (Thrombectomy Alone vs CDT) | | | |
|  | Average Treatment Effect | 95% lower bound | 95% upper bound | p-value |
| **Index Admission** | | | | |
| Died during hospitalization | 0.004 | -0.0001 | 0.009 | 0.056 |
| Discharge other than Home | 0.011 | -0.003 | 0.026 | 0.132 |
| GI Bleed | 0.002 | -0.003 | 0.007 | 0.461 |
| Intracranial Hemorrhage | 0.004 | 0.001 | 0.007 | 0.003 |
| Blood Transfusion | 0.012 | 0.006 | 0.019 | <0.001 |
| Total Cost | 0.086 | 0.068 | 0.104 | <0.001 |
| **6-month Readmission** | | | | |
| Unplanned Readmission | 0.014 | 0.0003 | 0.027 | 0.044 |
| GI Bleed | 0.004 | 0.0005 | 0.008 | 0.027 |
| ICH | 0.001 | -0.001 | 0.004 | 0.245 |
| Pulmonary Embolism | 0.010 | 0.002 | 0.018 | 0.020 |
| Blood Transfusion | 0.005 | 0.000 | 0.010 | 0.039 |
| Death at 6-months | 0.005 | 0.000 | 0.011 | 0.045 |

**Supplementary Table 6. Primary Diagnoses at First Unplanned Readmission Within 6 Months, by Treatment Group**

**Table A. Distribution of Primary Diagnoses by ICD-10 Chapter**

*Survey-weighted frequencies; ranked by CDT frequency.*

| **ICD-10 Chapter** | **CDT Weighted N** | **CDT %** | **MT Weighted N** | **MT %** |
| --- | --- | --- | --- | --- |
| I: Circulatory | 762 | 31.0 | 488 | 31.6 |
| A–B: Infectious | 231 | 9.4 | 208 | 13.5 |
| K: Digestive | 254 | 10.4 | 129 | 8.4 |
| J: Respiratory | 219 | 8.9 | 112 | 7.3 |
| S–T: Injury/Poisoning | 195 | 7.9 | 113 | 7.3 |
| C–D: Neoplasms/Blood | 172 | 7.0 | 147 | 9.5 |
| R: Symptoms/Signs | 145 | 5.9 | 58 | 3.8 |
| N: Genitourinary | 125 | 5.1 | 72 | 4.7 |
| E: Endocrine/Metabolic | 66 | 2.7 | 38 | 2.5 |
| G: Nervous System | 61 | 2.5 | 44 | 2.8 |
| F: Mental/Behavioral | 58 | 2.4 | 17 | 1.1 |
| M: Musculoskeletal | 57 | 2.3 | 36 | 2.4 |
| L: Skin | 55 | 2.2 | 23 | 1.5 |
| Other/Unknown | 38 | 1.5 | 46 | 3.0 |
| O: Pregnancy | 10 | 0.4 | 4 | 0.3 |
| Z: Factors/History | 4 | 0.2 | 7 | 0.4 |
| H: Eye/Ear | 2 | 0.1 | — | — |

**Table B. Top 10 Primary Diagnoses at First Unplanned Readmission — CDT Group**

| **ICD-10 Code** | **Diagnosis** | **Weighted N** | **%** |
| --- | --- | --- | --- |
| I2699 | Pulmonary embolism, unspecified | 169 | 6.9 |
| A419 | Sepsis, unspecified organism | 141 | 5.7 |
| J189 | Pneumonia, unspecified organism | 58 | 2.4 |
| I110 | Hypertensive heart disease with heart failure | 41 | 1.7 |
| N179 | Acute kidney failure, unspecified | 40 | 1.6 |
| I82412 | Acute embolism and thrombosis of right femoral vein | 34 | 1.4 |
| J9601 | Acute respiratory failure with hypoxia | 33 | 1.4 |
| N390 | Urinary tract infection, site not specified | 31 | 1.3 |
| I2692 | Saddle embolus of pulmonary artery with acute cor pulmonale | 31 | 1.3 |
| U071 | COVID-19 | 29 | 1.2 |

**Table C. Top 10 Primary Diagnoses at First Unplanned Readmission — MT Group**

| **ICD-10 Code** | **Diagnosis** | **Weighted N** | **%** |
| --- | --- | --- | --- |
| A419 | Sepsis, unspecified organism | 122 | 7.9 |
| I2699 | Pulmonary embolism, unspecified | 120 | 7.8 |
| U071 | COVID-19 | 46 | 3.0 |
| I2692 | Saddle embolus of pulmonary artery with acute cor pulmonale | 37 | 2.4 |
| I214 | Non-ST elevation (NSTEMI) myocardial infarction | 28 | 1.8 |
| I82412 | Acute embolism and thrombosis of right femoral vein | 24 | 1.6 |
| N179 | Acute kidney failure, unspecified | 24 | 1.6 |
| T83511A | Infection due to indwelling urethral catheter | 23 | 1.5 |
| J189 | Pneumonia, unspecified organism | 23 | 1.5 |
| J9601 | Acute respiratory failure with hypoxia | 23 | 1.5 |

Abbreviations: CDT, catheter-directed thrombolysis; MT, mechanical thrombectomy (thrombectomy alone); ICD-10, International Classification of Diseases, Tenth Revision; N, survey-weighted national estimate. Percentages reflect proportion of all first unplanned readmissions within each treatment group. COVID-19 diagnoses (U071) reflect patients admitted during 2020–2022.

**Supplementary Table 7. Inverse Probability Weighted Regression Adjustment (IPWRA) Results — High-Risk Pulmonary Embolism Subgroup**

*Thrombectomy alone vs. CDT; unweighted n = 2,081; survey-weighted N ≈ 3,945.*

| **Thrombectomy Alone vs. CDT — Average Treatment Effect (ATE¹)** | | | |
| --- | --- | --- | --- |
| **Outcome** | **ATE** | **95% CI** | **p-value** |
| **Index Admission** | | | |
| In-Hospital Mortality | -0.025 | −0.062 to 0.012 | 0.191 |
| Discharge Other than Home | +0.101 | 0.051 to 0.150 | <0.001 |
| GI Bleed | +0.003 | −0.018 to 0.024 | 0.760 |
| ICH | +0.008 | −0.008 to 0.024 | 0.314 |
| Blood Transfusion | +0.030 | 0.002 to 0.059 | 0.038 |
| Total Cost (ln-transformed) | +0.222 | 0.161 to 0.283 | <0.001 |
| **6-Month Follow-up** | | | |
| Unplanned Readmission | -0.027 | −0.069 to 0.016 | 0.226 |
| GI Bleed Readmission | +0.001 | −0.009 to 0.011 | 0.792 |
| ICH Readmission | +0.005 | −0.003 to 0.013 | 0.237 |
| PE Readmission | -0.037 | −0.059 to −0.014 | 0.002 |
| Blood Transfusion Readmission | +0.008 | −0.012 to 0.027 | 0.441 |
| Death at Final Follow-up | -0.020 | −0.058 to 0.018 | 0.309 |

¹ ATE: Average Treatment Effect of Thrombectomy Alone compared with CDT. Positive ATE = higher rate or cost with MT relative to CDT. Estimated using inverse probability weighted regression adjustment (IPWRA) with survey weighting (DISCWT). Propensity model included age, sex, Elixhauser comorbidity components, year, payer, and hospital characteristics. For Total Cost (ln-transformed), an ATE of 0.222 corresponds to approximately 24.8% higher costs with MT. High-risk PE defined as Massive_PE = 1 (presence of hemodynamic instability or cardiac arrest codes). ICH, intracranial hemorrhage; GI, gastrointestinal; PE, pulmonary embolism; CDT, catheter-directed thrombolysis; MT, mechanical thrombectomy; CI, confidence interval.
